# Dynamics of immunoglobulin sequence diversity in HIV-1 infected individuals

**DOI:** 10.1098/rstb.2014.0241

**Published:** 2015-09-05

**Authors:** Kenneth B. Hoehn, Astrid Gall, Rachael Bashford-Rogers, S. J. Fidler, S. Kaye, J. N. Weber, M. O. McClure, SPARTAC Trial Investigators, Paul Kellam, Oliver G. Pybus

**Affiliations:** 1 Department of Zoology, University of Oxford, Oxford, UK; 2 Wellcome Trust Sanger Institute, Cambridge, UK; 3 Cambridge Institute of Medical Research, University of Cambridge, Cambridge, UK; 4 Faculty of Medicine, Imperial College, London, UK; 5 MRC/UCL Centre for Medical Molecular Virology, Division of Infection and Immunity, University College London, London, UK

**Keywords:** B-cell receptor, diversity, Gini index

## Abstract

Advances in immunoglobulin (Ig) sequencing technology are leading to new perspectives on immune system dynamics. Much research in this nascent field has focused on resolving immune responses to viral infection. However, the dynamics of B-cell diversity in early HIV infection, and in response to anti-retroviral therapy, are still poorly understood. Here, we investigate these dynamics through bulk Ig sequencing of samples collected over 2 years from a group of eight HIV-1 infected patients, five of whom received anti-retroviral therapy during the first half of the study period. We applied previously published methods for visualizing and quantifying B-cell sequence diversity, including the Gini index, and compared their efficacy to alternative measures. While we found significantly greater clonal structure in HIV-infected patients versus healthy controls, within HIV patients, we observed no significant relationships between statistics of B-cell clonal expansion and clinical variables such as viral load and CD4^+^ count. Although there are many potential explanations for this, we suggest that important factors include poor sampling resolution and complex B-cell dynamics that are difficult to summarize using simple summary statistics. Importantly, we find a significant association between observed Gini indices and sequencing read depth, and we conclude that more robust analytical methods and a closer integration of experimental and theoretical work is needed to further our understanding of B-cell repertoire diversity during viral infection.

## 1. Introduction

The recent application of high-throughput sequencing technology to immunology, and to the characterization of B-cell and T-cell receptor diversity in particular, has the potential to reveal immune system dynamics in unprecedented detail [1–3]. This work has led to advances in our understanding of antibody dynamics after vaccination [4,5], the effects of aging and infection on the B-cell repertoire [6], the development of B-cell cancers [7,8] and the onset of autoimmune disorders such as multiple sclerosis [9]. Many of these studies have taken an ‘antibodyome’ perspective to viral infection, in which the immune response to infection is investigated by the bulk sequencing from patient samples of the antigen-binding regions of B-cell immunoglobulin (Ig) genes. These sequences are then analysed to explore how the antibody repertoire changes in response to perturbation arising from viral evolution and vaccination. This approach has been applied, for example, to influenza virus [5,10], varicella-zoster virus [11] and dengue virus [12].

Some of the most notable findings in this field have come from detailed investigation of the interactions between the humoral immune system and HIV-1 infection. Much of this work has focused on observing and understanding the development of broadly neutralizing antibodies (bNAbs) in order to aid the development of preventative HIV-1 vaccines [13]. Interestingly, bNAb precursors may be preferred in vaccine design over observed bNAbs because they may have a wider binding profile, and the sequences of such precursors can be inferred using methods of phylogenetic ancestral sequence reconstruction [14]. This requires an understanding of the diversity and dynamics of B-cell clones during infection, and significant strides have been made, both in tracking the coevolution of B-cell and viral lineages, [15–17] and in finding potential bNAb precursors using phylogenetic methods [14].

Despite these advances, more general trends in B-cell diversity and clonal dynamics during HIV-1 infection are still poorly understood, particularly during early infection. While some studies have contrasted the plasmablast, naive and memory B-cell content between early and chronic infections [18], the diversity of patient ‘antibodyomes' has not yet been characterized, either in comparison to healthy controls or through time in individuals that have recently seroconverted. Although early anti-retroviral therapy (ART) has been shown to have a significant effect on viral divergence (e.g. [19]), the corresponding effect on B-cell clonal diversity under ART is unknown. While it may be expected that B-cell populations in HIV+ individuals are more clonal than in uninfected individuals, and that the degree of B-cell clonal relatedness is related to the size of the concurrent viral population, these associations have not been explicitly tested.

Most previous studies that investigated the repertoire of B-cell receptor (BCR) diversity in peripheral blood (as opposed to those that focused on specific clonal lineages, e.g. [15]) have typically used non-phylogenetic approaches to summarize B-cell diversity. One successful approach has been to use single-linkage clustering to group sequences into clusters (or clones), and then to infer clonal expansions by measuring properties of the size distribution of clones using entropy scores, such as the Gini index [8]. Others studies have used alternative statistics, including mean clone size, the number of unique IgHV-D-J allele combinations, and genetic distances between IgV segment sequences and their respective germline homologues (e.g. [7]). Both sets of approaches have shown promise in studying B-cell cancers, which result from significant clonal expansions of usually one BCR lineage [20], but it is unknown if a similar approach will be informative when studying immune responses to HIV infection.

To better understand B-cell repertoire dynamics during early HIV infection and the degree to which it is modulated by ART, we used deep-sequencing to capture the diversity of Ig heavy-chain sequences from eight HIV patients enrolled in the short pulse anti-retroviral therapy at seroconversion (SPARTAC) trial [21]. This is, to our knowledge, the first time high-throughput BCR sequencing has been applied to studying general B-cell clonal diversity during ART and early HIV infection. Patients were enrolled an estimated 12–95 days after seroconversion, and were sampled at up to eight time points over approximately 2 years. At each time point, B-cell repertoire sequencing was performed and both viral load and CD4^+^ T-cell counts were measured. Three patients were untreated, while five received ART for the first 48 weeks of the study only. This study design not only allows us to track individual B-cell clones during early infection, but also to test for associations between the dynamics of B-cell sequence diversity and clinical variables, including treatment status.

For each patient and each time point, we used a high-throughput Illumina MiSeq platform to obtain paired-end reads from Ig heavy-chain sequences that represent the mixture of antibody classes in peripheral blood. Within each patient, we extended a previous single-linkage clustering approach [8] to classify sequences into clones and track their relative frequencies through time. We also explored a number of statistics in order to quantify BCR sequence diversity from HIV-1 infected patients, and to compare this diversity to that observed in a cohort of HIV-negative controls. While some general patterns were observed, overall we found a high degree of heterogeneity in B-cell clonal dynamics both among patients and through time.

## 2. Material and methods

### (a) HIV patients

Peripheral blood mononuclear cells (PBMCs) were isolated from eight patients with primary HIV-1 infection recruited from the SPARTAC study [21]. All had recently seroconverted before enrolment in the trial (an estimated 12–93 days before enrolment; median = 56 days). Patients 1–3 were untreated during the study period. Patients 4–8 received an ART regimen from week 0 to week 48, after which treatment was suspended. Patients were sampled between six and eight times over 108 weeks of the study (all time points are defined as weeks after start of the study, defined as week 0). All patients were sampled at weeks 4, 16, 24, 52, 60 and 108, whereas four patients were also sampled at week 0, and six at week 12. To ensure consistency among patients, and to ensure an equal number of time points during and after ART, only the former time points were used in analysis.

### (b) RT-PCR

RT-PCR reagents were purchased from Invitrogen and primers (supplied by Sigma Aldrich) are described by Van Dongen *et al.* [22] and in electronic supplementary material, table S1. Reverse transcription (RT) was performed using 500 ng of total PBMC RNA mixed with 1 μl JH reverse primer (10 μM), 1 μl dNTPs (0.25 mM) and RNase-free water added to make a total volume of 11 μl. This was incubated for 5 min at 65°C, and 4 μl First strand buffer, 1 μl DTT (0.1 M), 1 μl RNaseOUT™ Recombinant Ribonuclease Inhibitor and 1 μl SuperScript™ III reverse transcriptase (200 units μl^−1^) was added. RT was performed at 50°C for 60 min before heat-inactivation at 70°C for 15 min. PCR amplification of cDNA (5 μl of the RT product) was performed with the JH reverse primer and the FR1 forward primer set pool (0.25 μM each), using 0.5 μl Phusion^®^ High-Fidelity DNA Polymerase (Finnzymes), 1 μl dNTPs (0.25 mM), 1 μl DTT (0.25 mM), per 50 μl reaction. The following PCR programme was used: 3 min at 94°C, 35 cycles of 30 s at 94°C, 30 s at 60°C and 1 min at 72°C, with a final extension cycle of 7 min at 72°C on an MJ Thermocycler.

### (c) Sequencing and reference-based V-D-J assignment

MiSeq libraries were prepared using Illumina protocols and sequenced by 150 bp paired-ended MiSeq (Illumina). MiSeq reads were filtered for base quality (median more than 32) using QUASR (http://sourceforge.net/projects/quasr) [23]. Sequences were concatenated and a gap inserted between the forward and reverse reads (average gap length approx. 35 nucleotides; electronic supplementary material, figure S2). Non-Ig sequences were removed; only those reads with significant similarity to reference IgHV and IgHJ genes from the ImMunoGeneTics (IMGT) database [24] were retained, as determined using BLAST [25] with *E*-value thresholds of 1 × 10^−10^ and 1 × 10^−3^_,_ respectively. Primer sequences were then trimmed from the reads, and sequences retained for analysis only if both primer sequences were identified and if both forward and reverse sequence lengths were more than 100 bp. Lastly, a second BLAST analysis was used to identify the IgHV genes of retained reads. Specifically, BLAST [25] was used to align reads against known BCR sequences from the IMGT database [24] (*E*-value thresholds for IgHV and IgHJ genes were 10^−70^ and 10^−20^, respectively, due to their different gene lengths). The combined per-base error rate for the RT-PCR and sequencing process of the MiSeq platform was 2.06 × 10^−4^ [8,26]. Sequences and BAM files are available from the European Nucleotide Archive under study accession no. ERP000572.

### (d) Alignment

The data comprised non-overlapping paired-end reads separated by a gap of variable and unknown length. This necessitated certain heuristic measures during data processing and the two reads of each pair were aligned separately to ensure positional homology. As above, reference IGHV and IGHJ segments were obtained from IMGT [24]. Gaps were removed from the reference sequences, then IGHV sequences were clustered using CD-HIT [27] with a 95% similarity threshold. Only one sequence was retained from each cluster, resulting in a smaller set of V segments. The IHGJ reference sequences were not down-sampled, however. The first read of each pair was aligned with the closest matching sequence in the V references through gapless pairwise alignment. To further optimize the alignment, each read was matched using only the 5′ half of the V segments. Next, the second read of each pair was pairwise aligned with the closest sequence in the J references, again using gapless alignment. Once the first and second reads had been aligned to the V and J references, respectively, gaps were added 3′ of the first read, and 5′ of the second read, such that each were 300 nucleotides in length. The two reads were then concatenated, and excess gaps were trimmed between each read pair. A graphical representation of this process is provided in electronic supplementary material, figure S3. Because we expect sequences from the same clone to match the same, or highly similar, V and J segments, this process generates an approximation of the optimal gapless alignment for sequences from the same clone. Further, the process does not require sequences to match the same V and J segments in order to be compared, a requirement that may be problematic when dealing with highly mutated sequences. However, the process may produce sub-optimal alignments when indel diversity is present within a clone. A small percentage of reads showed high similarity between forward and reverse reads (longest common sub-sequence of 20 bp or greater) and were excluded during this step.

### (e) Clonal assignment

Sequences from all time points within each patient were pooled and assigned to clones using single-linkage agglomerative clustering using the Hamming distance between two sequences [8], while ignoring gap variation. This latter condition was necessary because each aligned sequence had an arbitrary gap between its two reads that resulted from paired-end sequencing, and not from a natural process of immunological diversification. To make our results comparable with previous work [8], we also chose a maximum Hamming distance of unity for clustering two sequences together into the same clone.

### (f) Measurement of B-cell receptor diversity

**Figure 1.**
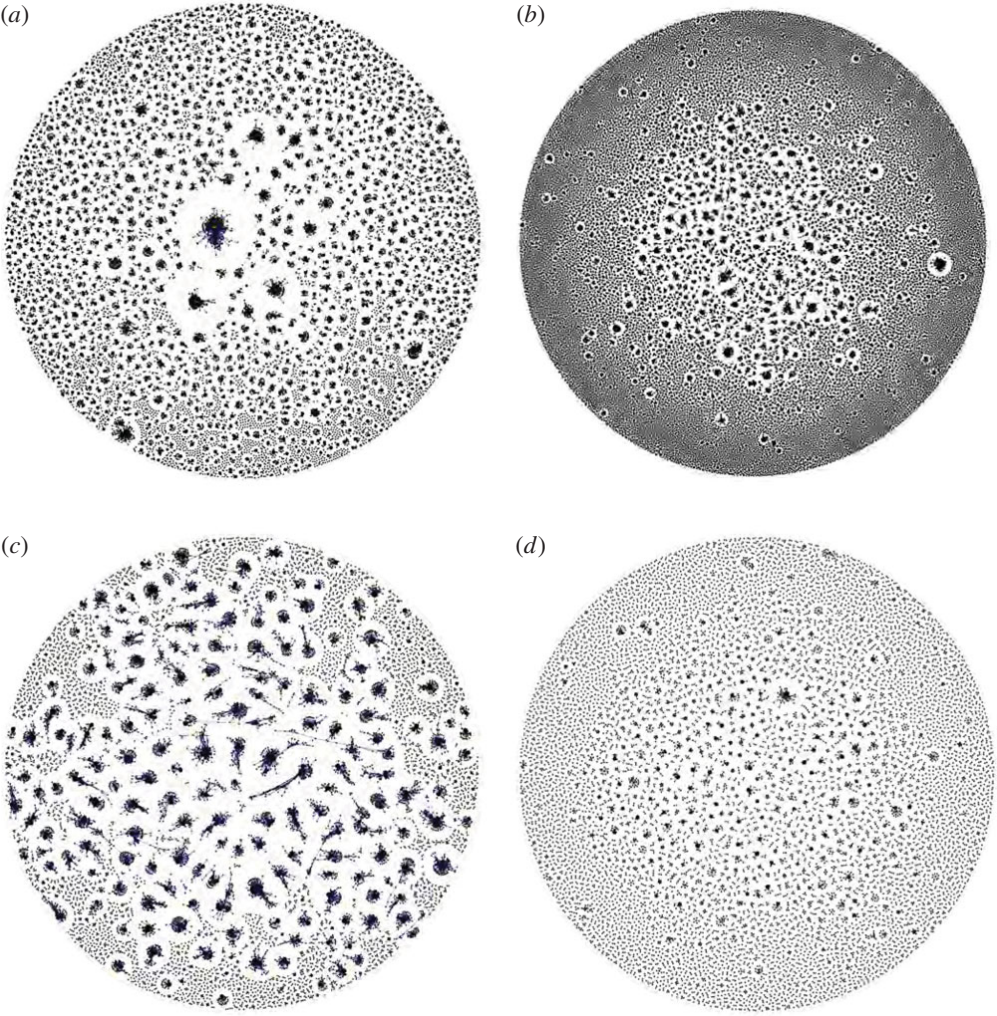
Network visualization of the diversity of BCR sequences obtained from untreated patient 3 at week 4 (*a*) and week 108 (*b*), and from treated patient 4 at week 4 (*c*) and week 108 (*d*). Each vertex represents a unique sequence, and the size of each vertex is proportional to the number of reads identical to that sequence. Edges are drawn between vertices that are one nucleotide substitution apart. See §2(f) for further details.

The diversity of BCR sequences was qualitatively visualized using the network approach developed by Bashford-Rogers *et al.* [8]. Briefly, each vertex represents a unique BCR sequence, whose relative size is proportional to the number of sequence reads identical to the vertex sequence. Edges are then drawn between vertices whose sequences differ by at most one nucleotide change. Networks were computed using igraph, as implemented in R (http://igraph.sourceforge.net; see [8] for details). Because each clone is shown in proportion to its relative frequency in the BCR sequence population, these networks provide an intuitive visualization of the clone size distribution. Examples of these plots from representatives of the treated and untreated patient groups are shown in figure 1.

Three sample summary statistics were applied to the BCR sequences obtained at each time point to summarize their clonal diversity: (i) mean clone size, (ii) vertex Gini index, and (iii) the proportion of reads in ‘large clones’. Each of these three statistics is described below.

For each time point and each clone, clone size was defined as the number of reads in the clone at that time point divided by the total number of reads at that time point. Mean clone size was calculated as the arithmetic mean of this distribution of clone sizes. Next, the vertex Gini index was used to summarize inequality in BCR sequence frequency; the index equals unity if all reads have the same BCR sequence (maximum inequality), and equals zero if each BCR sequence is equally common (maximum equality). As defined above, vertex size equals the number of identical reads with a unique BCR sequence, so the vertex Gini index was computed directly from the vertex size distribution as formed using a clustering cut-off of zero. Bashford-Rogers *et al.* [8] explored both the vertex and cluster Gini indices, and found that the former correlated better with clinical parameters in chronic lymphocytic leukaemia (CLL) patients; hence only the vertex Gini index is used here.

Clones were classified as ‘large clones' if they comprised at least 0.1% of the reads sequenced at *any* of the time points in which they were found. We quantified these by calculating the proportion of reads at each time point that belong to ‘large clones'. Additionally, we wished to describe changes in the very upper tail of the clonal size distribution. To do so, we plotted the sizes of the 20 largest clones as a proportion of the total number of reads at each time point. These values are for illustrative purposes only and are not used as sample statistics.

### (g) Sub-sampling

Preliminary statistical analysis using ANOVA showed that Gini index values were significantly associated both with patient identity (*p* = 0.02) and read depth (*p* = 0.001). A plot of Gini index values against read depth for each time point highlights the variation in read depth among patients (electronic supplementary material, figure S4). To ensure that comparisons among patients are reliable and not an artefact of read depth variation, all further analyses were undertaken on subsamples of the original data. Specifically, 70 000 sequences were randomly sampled from each time point in each HIV+ patient (the lowest number of reads available for a HIV+ patient sample was 74 861). When comparing HIV+ patients with healthy controls (see §2i), then 5000 sequences were randomly sampled from each control and HIV+ dataset (the lowest number of reads available for a control patient was 5087). In each case, random sub-sampling of reads was repeated 10 times. As variance in BCR statistics among these 10 repetitions was very low (less than 1 × 10^−4^), we only report the mean values of the 10 sub-sampling repetitions.

### (h) Analysis of B-cell receptor diversity

To examine the relationship between BCR sequence diversity and clinical variables, we compared each of the above BCR statistics to log(viral load) and CD4^+^ counts using linear regression. *p*-values were adjusted for multiple hypothesis testing using the Benjamini–Hochberg procedure [28] (as implemented in the R function p.adjust). To account for possible temporal autocorrelation within patients, all data points within each patient were summarized using two means. Because ART will have a significant effect on many variables, such as viral load, we calculated separate means for values from early time points (weeks 4–24; i.e. during ART for treated patients) and from late time points (weeks 52–108; when no patients were receiving ART). These means were calculated by linearly interpolating between adjacent time points, and then calculating the mean of the resulting function between the first and last points (weeks 4 and 24 for early only, 52 and 108 for late only). ANOVAs were used to explore how the Gini index and other BCR summary statistic values vary among patients, time points, treatment status and read depth. To make linear regression coefficients of each regression more comparable, all variables besides viral load and CD4^+^ count were scaled to a mean of zero and variance of unity before linear regression and ANOVA analysis.

### (i) Comparison with healthy controls

To test whether BCR sequence diversity differs between HIV-infected and uninfected individuals, we analysed previously published data from a cohort of six healthy patients [26]. For each, non-gapped Illumina MiSeq reads were obtained from PBMCs. See [26] for full details of RNA capture, PCR and sequencing for the healthy controls. To ensure that these non-gapped sequences are directly comparable to the gapped sequences obtained from HIV patients, we only used the first and last 110 bp of each read, placing a gap of unknown length between. Because these patients were sequenced at a lower read depth (5087–8475 reads) than HIV+ patients, all datasets from healthy controls were randomly sub-sampled (as described in §2g) to remove any potential bias arising from variable read depth. Further, when comparing with healthy controls, only within-time point edges were allowed when identifying clones in HIV patients (because sequences from only one time point were available from the control individuals). Differences in BCR diversity statistics between control and HIV-infected individuals were tested using Wilcoxon tests.

## 3. Results

Figure 1 provides a qualitative visualization of BCR sequence diversity, and its change between the beginning and end of the study period (weeks 4 and 108, respectively). The data shown are from patients 3 and 4 and the plots were computed using the network approach developed in [8]. For both patients, the sequences at week 4 are clearly more clonally clustered compared with those at week 108, although the BCR diversity for patient 4 (figure 1*c*) appears more polyclonal than for patient 3 (figure 1*a*) at this time. As expected, at neither time point is the BCR population dominated by a single large clone, as was often observed in samples from B-cell lymphoma patients [8]. The plots shown in figure 1 are largely typical of those observed for other patients.

**Figure 2.**
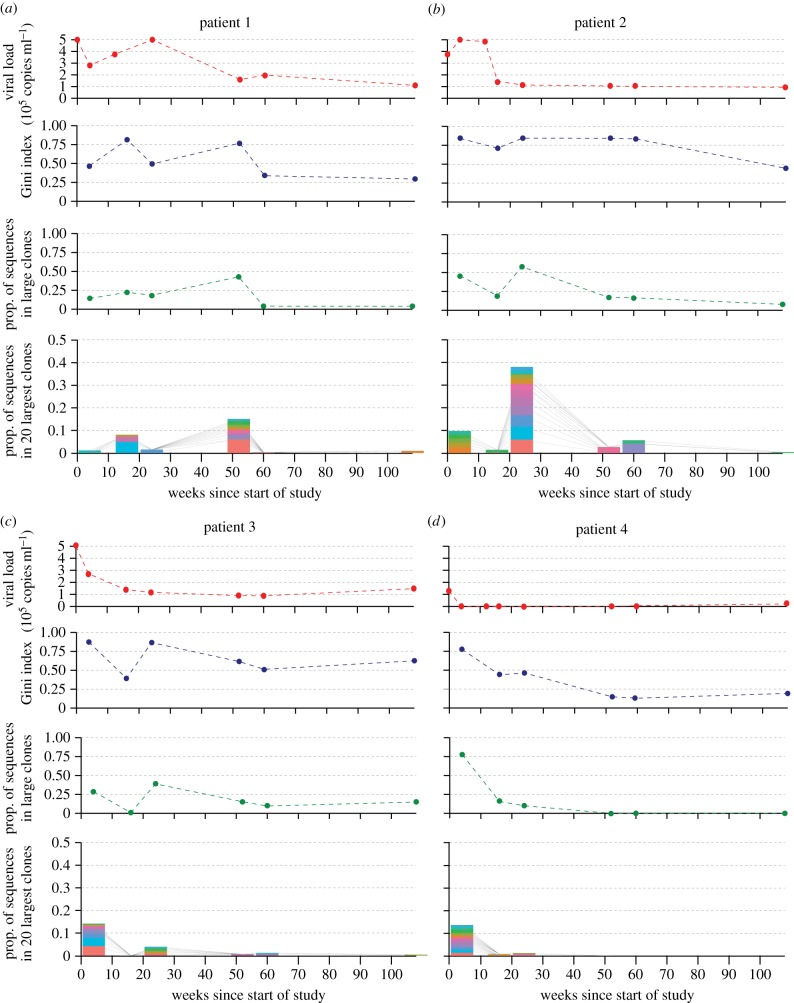
BCR diversity statistics and viral load values for patients 1–4 (*a*–*d*) over the study period of 108 weeks. Patients 1–3 were untreated, while patient 4 received ART until week 48. Four plots are provided in each panel. The top plot shows viral load, with each point representing a clinical sample. The second plot shows vertex Gini index values of the BCR sequences obtained at each time point. The third plot shows the proportion of reads at each time point that belong to ‘large’ clones, i.e. those that occupy more than 0.1% of reads at any time point. The bottom plot in each panel shows the proportion of all reads at each time point that are occupied by the 20 largest clones observed across all time points. Each of the 20 largest clones is represented by a bar of a different colour, and lines connect bars at adjacent time points that represent the same clone.

**Figure 3.**
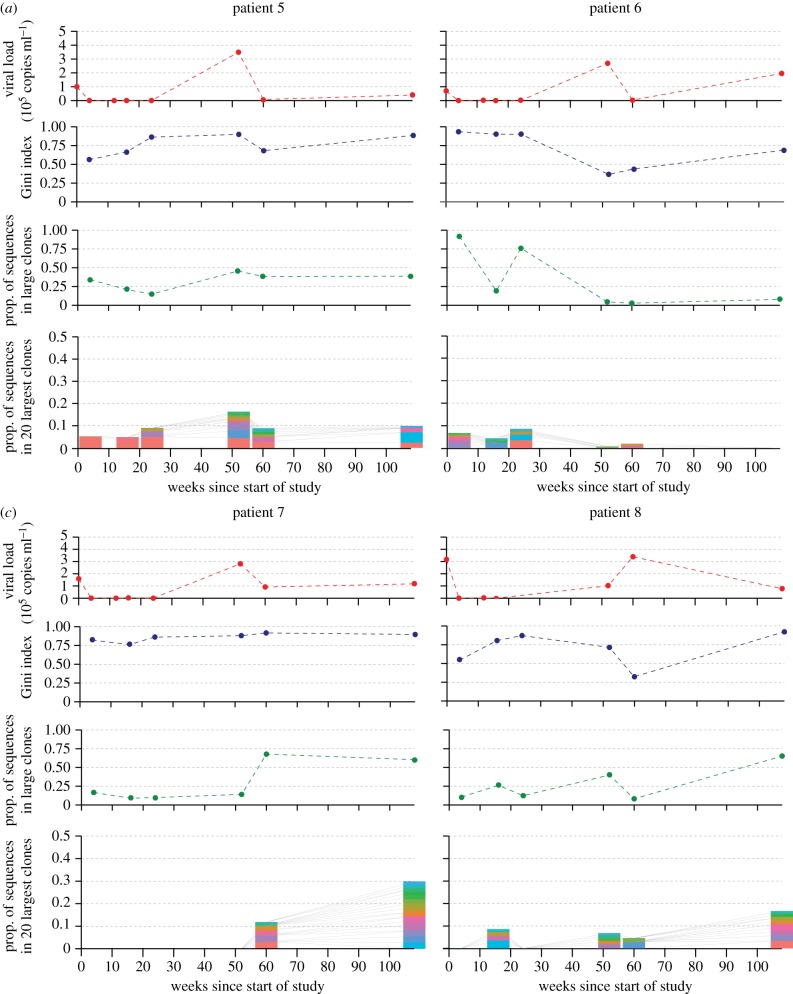
BCR diversity statistics and viral load values for patients 5–8 (*a–d*) over the study period of 108 weeks. All patients received ART until week 48. See figure 2 legend for details.

Full results from all patients are summarized in figures 2 and 3. All patients had seroconverted recently (median = 56 days) before the start of the study (week zero). Patients 1–3 were untreated through the course of the study period (weeks 0 to 108). Patients 4–8 received an ART regimen between weeks 0 and 48, after which they were untreated. BCR sequencing was performed on PMBC samples from each time point for each patient (yielding 7.4 × 10^4^ to 1.0 × 10^6^ filtered BCR reads per sample). BCR network analysis was applied to these sequencing datasets to quantify the clonal architecture of these samples according to Bashford-Rogers *et al*. [8]. Four plots are shown for each patient, which show the change through time in (i) viral load, (ii) the vertex Gini index of BCR sequences, (iii) the proportion of BCR sequences in ‘large clones', and (iv) the size distributions of the 20 largest clones as a proportion of the total number of reads at each time point.

### (a) Untreated patients

All three untreated patients (1–3) showed peak viral loads at or within four weeks of enrolment, and reduced, but persistent viraemia after 52 weeks corresponding to set point viral load. Patient 1 had an additional peak in viral load at 24 weeks, suggesting a superinfection event, and showed a higher viral load for a longer duration than patients 2 and 3. In all untreated patients, Gini indexes and the proportion of reads in large clones were typically higher during the first half of the study, when viral loads were higher. Hence at these times, a higher proportion of the BCR sequence population was composed of common sequences/clones. When ‘large clones' dominated the BCR population (e.g. week 24 in patient 2), that dominance was not the result of a single dominant clone, but rather resulted from the concurrent increase in frequency of a large number of different clones (see bottom plot of each panel in figure 2). Further, the relative frequencies of individual clones that were found at high frequencies (colours in bottom plot of each panel in figure 2) fluctuated through time with no discernable pattern; in almost all cases, clones were common at only one time point.

### (b) Treated patients

All five treated patients (4–8) showed a rapid decrease in viral load soon after the start of the study, with counts in weeks 16–24 below 100 copies ml^−1^ (often less than 50, or undetectable), coinciding with the period of ART. As expected, viral load values across all time points were significantly lower in the treated group than the untreated group (*one sided t-test*; *p* < 0.0001). Patients 5–8 showed a rebound in viral load at week 52, after the cessation of ART, while the rebound in patient 4 was not as strong and viral loads remained relatively low (less than 21 000 copies ml^−1^) throughout the study period. In patients 5–7, viral load decreased in week 60 before stabilizing at 108 weeks at a similar level to that in untreated patients.

The dynamics of B-cell diversity and clonality in the treated patients were highly variable. Patient 7 showed a striking pattern after cessation of ART: at week 60, there was a polyclonal expansion (such that most BCR sequences fell into ‘large’ clones) which remained in week 108. Patient 8 also showed polyclonal responses and an increase in the proportion of sequences in large clones at weeks 52 and 108.

BCR diversity dynamics in patient 6 in many ways resembled those of untreated patients 2 and 3, with a peak in the prevalence of ‘large clones' in week 24, and low clonality in week 108. Patient 4 exhibited high viral load and clonality at week 4, after which both remained low for the rest of the study. Patient 5 showed a comparatively stable level of clonality through time, with the proportion of sequences in large clones ranging from 0.13 to 0.45. Notably, patient 5 is the only patient to exhibit a ‘large clone’ that persisted throughout the study period (red bars in the bottom panel of figure 3*a*). Across time points, this persistent clone occupied between 2.4 and 5.1% of all BCR sequences.

### (c) Relationship between clinical variables and B-cell diversity

We sought to test whether statistics of BCR diversity were associated with clinical variables, such as viral load, time since seroconversion and CD4^+^ cell count (electronic supplementary material, figure S6). To enable direct comparisons among patients, all datasets were sub-sampled to exactly 70 000 reads (see §2g). For each patient, mean values during the early and late periods were used to account for autocorrelation (see §2h). As shown in table 1, there was no significant association between log viral load, CD4^+^ cell count, or estimated days since seroconversion with any of the three BCR diversity statistics. In all cases, correlations were non-significant both before and after correction for multiple hypothesis testing. The results also remained non-significant when repeated without adjusting for temporal autocorrelation (i.e. each time point in each patient counted as an independent data point).

**Table 1.** Regression analysis of BCR diversity statistics and clinical variables.

clinical variable	BCR diversity statistic	correlation	adjusted *p*-value^a^
log(viral load), early period	mean clone size	−0.005	0.99
	Gini index	−0.13	0.92
	proportion of reads in large clones	−0.19	0.92
log(viral load), late period	mean clone size	0.3	0.92
	Gini index	0.37	0.92
	proportion of reads in large clones	0.28	0.92
CD4 count, early period	mean clone size	−0.3	0.92
	Gini index	−0.43	0.92
	proportion of reads in large clones	0.13	0.92
CD4 count, late period	mean clone size	−0.34	0.92
	Gini index	−0.47	0.92
	proportion of reads in large clones	−0.34	0.92
days from seroconversion at week 0, early period	mean clone size	−0.17	0.92
	Gini index	−0.19	0.92
	proportion of reads in large clones	0.25	0.92
days from seroconversion at week 0, late period	mean clone size	−0.29	0.92
	Gini index	−0.36	0.92
	proportion of reads in large clones	−0.21	0.92

^a^*p*-values were adjusted using the Benjamini–Hochberg procedure [28].

### (d) Analysis of B-cell receptor diversity and sub-sampling

To explore possible sources of variation in the three BCR statistics, we used ANOVAs to test whether each statistic was associated with patient identity, week of sampling and treatment group at each time point (table 2). All datasets were sub-sampled to 70 000 reads. None of these factors explained a significant proportion of variation in any of the BCR diversity statistics. Thus, the association between Gini index and patient identity observed prior to sub-sampling (see §2g and electronic supplementary material, figure S4) was most likely an artefact due to variation in read depth among patients.

**Table 2.** ANOVA analysis of BCR diversity statistics.

response	factor	mean square	*p*-value^a^
mean clone size	patient	0.99	0.63
	week	0.49	0.63
	treatment	0.6	0.63
Gini index	patient	1.7	0.57
	week	2.3	0.57
	treatment	0.2	0.74
proportion of reads in large clones	patient	0.48	0.87
	week	0.7	0.63
	treatment	1.1	0.63

^a^*p*-values were adjusted using the Benjamini–Hochberg procedure [28].

**Figure 4.**
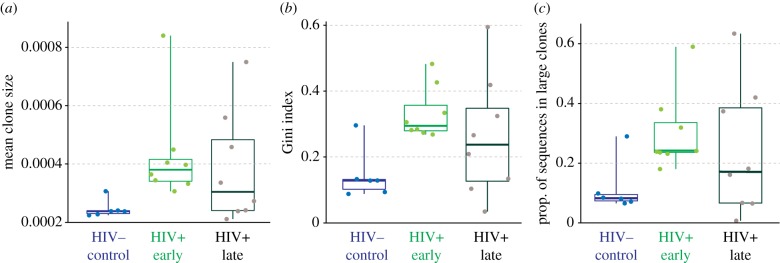
Comparison of three BCR summary statistics among healthy uninfected individuals (blue) and HIV-infected patients. Values from HIV+ patients are calculated separately for weeks 4–24 (HIV+ early; green) and weeks 52–108 (HIV+ late; black). For each period, statistics were averaged using linear interpolation (see §2f for details). Each box and solid line represents the first, third and second quartiles, respectively, while whiskers indicate range. Data points are superimposed on the box and whisker plots.

We also explored the effects of sub-sampling on each of the three BCR diversity statistics (electronic supplementary material, figure S7). As might be expected, the ‘proportion of reads in large clones' statistic was almost unaffected by sub-sampling. Although vertex Gini indices were slightly higher before sub-sampling, this inflation was relatively constant within patients. Mean clone size was substantially altered by changes in read depth, justifying our use of sub-sampling when comparing data from different patients (tables 1 and 2; figure 4).

### (e) Comparison with healthy controls

To enable direct comparisons between HIV+ patients and healthy controls, all datasets were sub-sampled to 5000 reads (see §2g). As before, values from HIV+ patients were grouped into two periods, early (weeks 4–24) and late (weeks 52–108) to avoid temporal autocorrelation. Comparisons of BCR sequence diversity are shown in figure 4. All three statistics (mean clone size, Gini index and the proportion of reads in large clones) were significantly higher in early-period HIV+ patients than in healthy controls (*p* = 0.01, 0.02 and 0.03, respectively). However, all comparisons between healthy controls and late-period HIV+ patients were non-significant.

## 4. Discussion

The results presented here demonstrate that BCR sequence diversity during HIV infection is highly dynamic and heterogeneous, both within and among patients. Individual clones fluctuate substantially in relative frequency, and at no time point did a single clone dominate the BCR sequence population in any patient. This suggests an actively evolving antibody response against HIV, likely due to the high turnover of HIV genetic diversity within each patient generating novel antigenic targets (e.g. [29]).

We observed significantly greater clonal structure in HIV+ patients than in uninfected controls during the first half of the study period, but not during the second half. This is consistent with a pronounced humoral response to viraemia during early infection. BCR diversity statistics during the late period were highly variable, suggesting that strong clonal structure was maintained in some patients but lost or significantly reduced in others (figure 4). However, when comparing HIV+ patients, we found no significant association between BCR diversity statistics and clinical variables, such as viral load or CD4^+^ count, suggesting a highly complex relationship between the adaptive immune system and viral dynamics. Previous studies have shown highly heterogeneous B-cell clonal responses to immune perturbations such as vaccines over a timescale of weeks [4], so the variation observed here over a period of approximately 2 years fits with established findings. Further, once differences in read depth were accounted for, BCR diversity statistics did not vary significantly with treatment group, time of sampling or patient identity.

There are several possible explanations for the heterogeneity we observed among HIV+ patients and through time. The first and simplest explanation is a lack of statistical power and sampling resolution. With only eight patients, the power of our study is almost certainly constrained by small sample size. However, a previous study of B-cell lymphoma patients that had a similar sample size and which used similar analytical techniques was able to establish significant associations between BCR clonality and clinical outcome in CLL patients, likely due to the exceptionally large B-cell clones generated by this cancer [8]. Another potentially important factor in our study is the coarseness of temporal sampling relative to the dynamics of the BCR sequence population. Each patient was sampled six to eight times over 2 years, with all time points at least four weeks apart. Further, the earliest sample taken after ART ended in the treated group was obtained in week 52. This is potentially significant because recent studies of vaccine responses that sampled more intensively have shown that B-cell clones originating at vaccination tend to expand within the first week after inoculation and afterwards rapidly contract, with little persistent expansion after four weeks [4]. Though we might expect clonal responses to chronic viral infection to be more persistent than those to vaccination, our sampling scheme (which was not initially designed with B-cell dynamics in mind) may not reveal consistent trends in BCR clonality during HIV infection. Fluctuations that might be associated with acute infection may be difficult to discern because (i) some patients were given early treatment, (ii) the time between seroconversion and the start of the study varied substantially (12–95 days), and (iii) in one patient there was evidence of HIV-1 superinfection during the study period. Further, BCR diversity changes associated with viral rebound after cessation of ART may not be apparent because patients were sampled several weeks later.

There are also biological explanations for absence of significant relationships between clinical variables and BCR diversity scores. While the BCR metrics used here were informative when applied to B-cell lymphoma patients, those patients often exhibited extreme clonality, such that one or a few B-cell clones comprised the majority of BCR reads [8]. However, the response of the BCR sequence population to viral infection is likely to be far more subtle and diverse. Because clonal expansion during infection is related to binding affinity to viral epitopes, BCR diversity will be related not only to the amount of virus present at the time of sampling, but also to the antigenic diversity of the virus population, B-cell longevity and complex ecological dynamics arising from antigenic cross-reactivity [17]. Further, the dynamical interaction between viraemia, helper T-cell counts and T-cell activation of HIV-specific B cells may further obscure relationships between BCR diversity and other measurements. The network visualizations (figure 1) and BCR statistics (figures 2 and 3) reported here indicate that the BCR clone size distribution during early HIV infection is much less skewed than that observed for B-cell cancers [8], meaning that HIV-specific clonal expansions may be difficult to detect from bulk antibody samples without some form of functional filtering or screening (e.g. antigen-specific cell sorting or isolation of plasmablasts [30]). If so, then further progress will require very close collaboration between experimental researchers and theoreticians interested in developing new analytical approaches. Another process of relevance to studies of HIV infection is B-cell hypermutation, which has been shown to generate substantial intra-clonal sequence diversity and divergence through time [14,15]. If high rates of hypermutation are common, then the conservative clustering algorithm used here (which only links sequences that differ by one mutation) may split large and diverse lineages into distinct groups, thereby reducing its ability to capture BCR dynamics. Further work is needed to discriminate between inter- and intra-clonal diversity in a statistically rigorous manner.

Our results are more successful in highlighting the potential usability and performance of different statistics of BCR diversity. Although the vertex Gini index proved informative in prior work [8], we found that it is positively correlated with sequencing read depth (electronic supplementary material, figure S4). Application of the Gini index to simple hypothetical datasets demonstrates why this correlation may arise (see electronic supplementary material, table S5). In short, as read depth increases, large clones grow in size yet more unique singleton reads are also added, thereby increasing the skew of the clone size distribution, resulting in a higher Gini index. Bashford-Rogers *et al.* [8] also explored the dependence of the Gini index on read depth, via random sub-sampling of empirical data (electronic supplementary material figure S9 in [8]), although in their study the effect was small compared with the difference between case and control patients. In analyses of HIV infection, meaningful differences in Gini indices are likely more subtle and thus this statistic may be less effective. One BCR statistic used here, the ‘proportion of reads in large clones', showed no significant relationship with read depth and was unaffected by sub-sampling (electronic supplementary material, figure S7) and is thus worth considering further. Importantly, while the Gini index attempts to characterize the entire clone size distribution, and is thus affected by changes in the relative frequency of very rare variants, statistics such as the proportion of reads in ‘large clones' focus on the dynamics of large clones. Substantial theoretical work is needed to understand the sampling properties of BCR diversity statistics and to develop more powerful alternatives to those used to date. It is likely that multiple informative statistics will be needed to adequately summarize BCR repertoires obtained through bulk sequencing, and may need to consider properties of the data other than the distribution of clone sizes.

Further methodological challenges face the analysis of BCR repertoire data obtained through bulk sequencing. A formal, phylodynamic analysis of BCR sequence data is appealing because it would allow for model-based inference of parameters such as hypermutation rates and the relative growth rates of clonal populations. However, because BCR lineages are created through somatic V(D)J recombination, bulk BCR sequences do not coalesce to a single common ancestor and must be separated into distinct lineages before analysis can proceed. Further, probabilistic models of sequence evolution will be computationally very demanding when applied to BCR datasets, some of which—including those used here—contain millions of unique sequences per patient. If they are developed in future, phylodynamic methods may be best applied to individual B-cell lineages that have been isolated or identified by experimental, rather than computational, methods.

## Supplementary Material

Supplementary Information
