# Supplementary material for: Dynamics of immunoglobulin sequence diversity in HIV-1 infected individuals
Source: Philos Trans R Soc Lond B Biol Sci. 2015 Sep 5;370(1676):20140241. doi: 10.1098/rstb.2014.0241 (PMC4528418; doi:10.1098/rstb.2014.0241)
Supplement: Supplementary Information [file rstb20140241supp1.pdf]

**Dynamics of immunoglobulin sequence diversity in HIV-1 infected individuals**

Kenneth B Hoehn, Astrid Gall, Rachael Bashford-Rogers, Fidler SJ, Kaye S, Weber JN, McClure MO, SPARTAC Trial Investigators, Paul Kellam, Oliver G Pybus

**Supplemental information**

S1: Primer sequences used
S2: Graphical outline of PCR
S3: Graphical outline of analysis pipeline
S4: Relationship between Gini index and read depth between patients
S5: Gini index and read depth example
S6: CD4+ cell counts and seroconversion information
S7: Effect of sub-sampling on BCR statistics

**S1: Human B-cell receptor PCR primers**

| Primer | Sequence | |
|---|---|---|
| JH reverse | CTTACCTGAGGAGACGGTGACC | |
| VH1-FR1 forward | GGCCTCAGTGAAGGTCTCCTGCAA G | |
| VH2-FR1 forward | GTCTGGTCCTACGCTGGTGAAACCC | |
| VH3-FR1 forward | CTGGGGGGTCCCTGAGACTCTCCTG | FR1 primer set* |
| VH4-FR1 forward | CTTCGGAGACCCTGTCCCTCACCTG | |
| VH5-FR1 forward | CGGGGAGTCTCTGAACATCTCCTGT | |
| VH6-FR1 forward | TCGCAGACCCTCTCACTCACCTGTG | |

**S2: Illustration of the BCR sequencing pipeline**

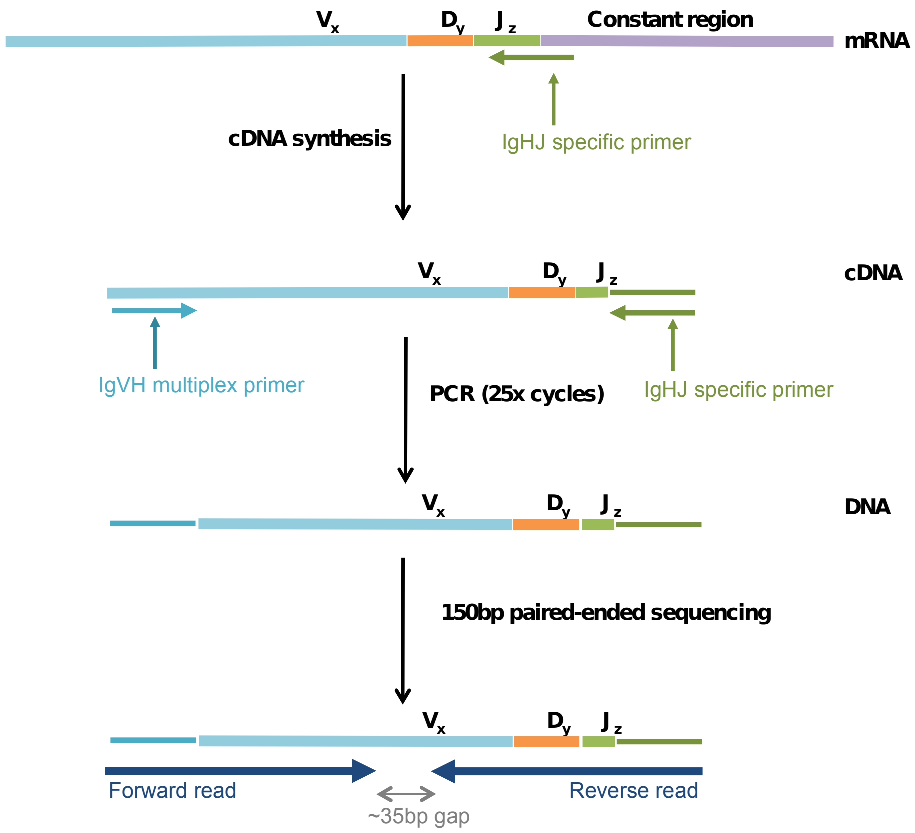

**S3: Illustration of the sequence alignment pipeline**

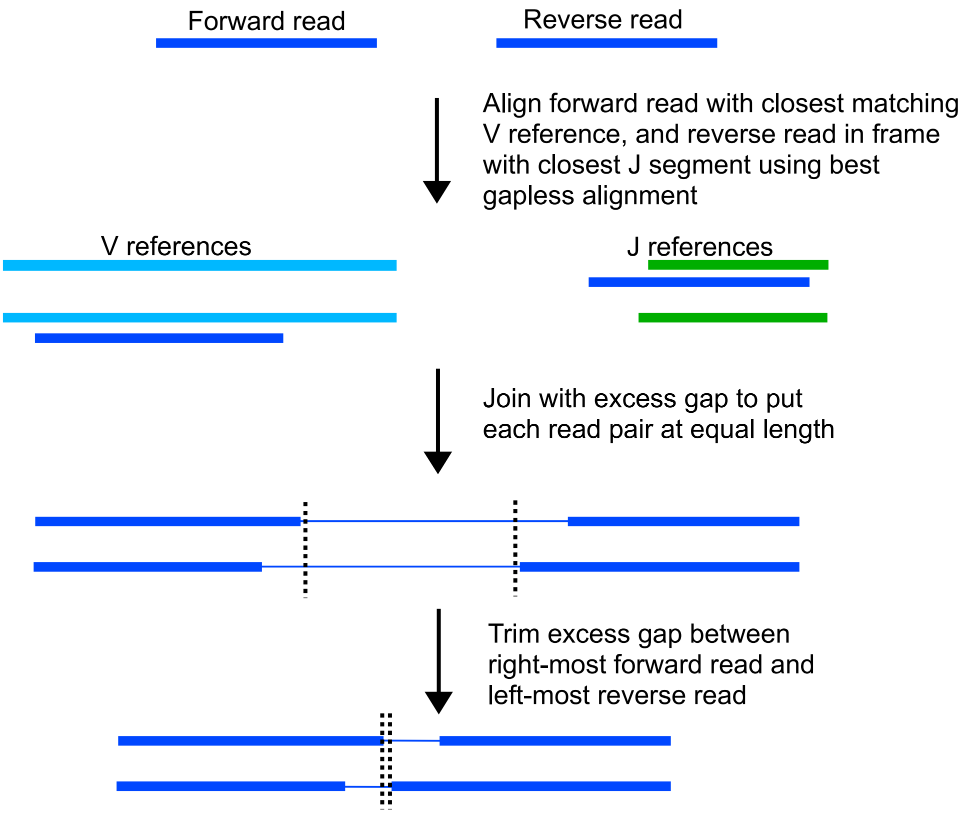

**S4: Relationship between Gini index and read depth between patients**

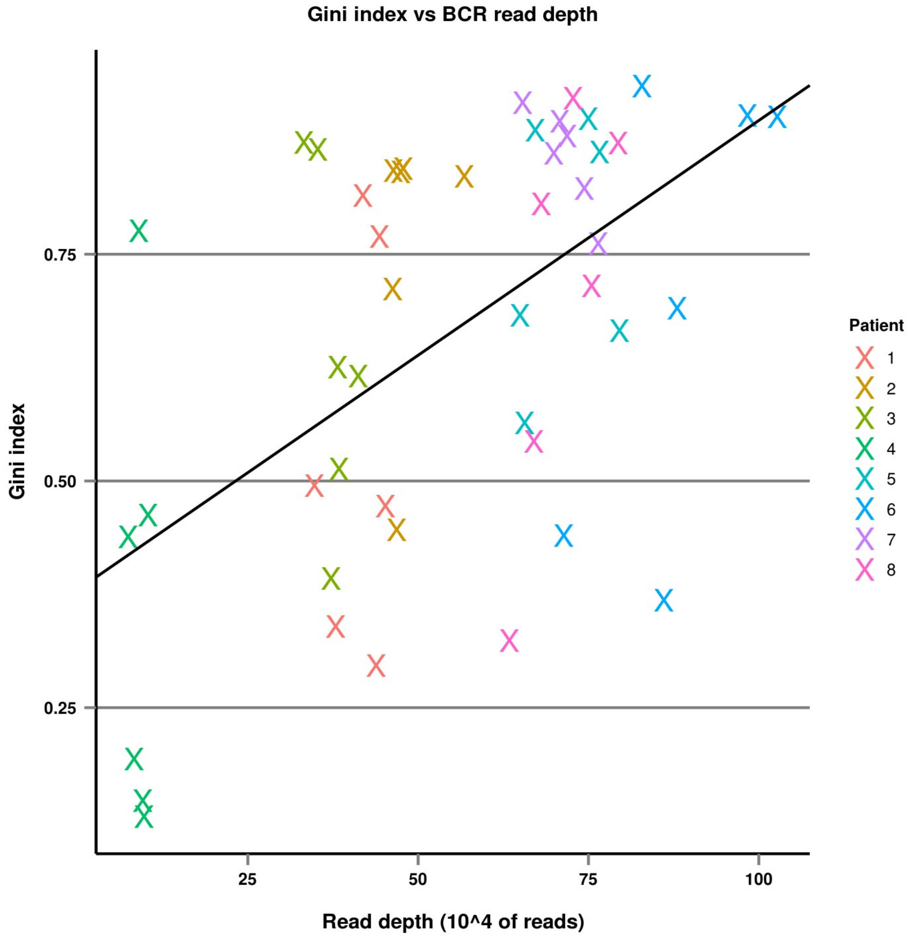


S4: Each point represents a sampling time point and is coloured by patient (see key). The fitted linear regression has a slope of $5.2x10^{-3}$ ($p = 5.69x10^{-5}$) and a correlation coefficient of 0.55. This relationship remained significant when patient means were used to correct for temporal autocorrelation ($p < 0.03$).

**S5: Gini index and read depth**

We model the effect of read depth on Gini index using a simple population of B cells consisting of a single dominant clone – occupying one third of all B cells – and a large pool of unique B cells, which comprise the rest of the system. As sequencing depth increases, one third of each sample joins the dominant clone, which grows larger. However, all other clones remain unique, leading to a larger population of singleton clones. This means the ratio of reads in the dominant clone to each of the singletons grows with sampling depth, leading to a more skewed size distribution and higher Gini index. Specific values are provided in the table below.

| Depth (total reads) | Largest clone (reads) | No. of Singletons | Gini index |
|---|---|---|---|
| 15 | 5 | 10 | 0.24 |
| 30 | 10 | 20 | 0.29 |
| 45 | 15 | 30 | 0.30 |
| 60 | 20 | 40 | 0.31 |
| 75 | 25 | 50 | 0.31 |

**S6: CD4+ cell counts and seroconversion information**

| | | CD4+ counts | | | | | |
|---|---|---|---|---|---|---|---|
| *Patient* | *Estimated days since seroconversion at week 0* | *Week 4* | *Week 16* | *Week 24* | *Week 52* | *Week 60* | *Week 108* |
| *1* | 95 | 470 | NA | 440 | 480 | 50 | 470 |
| *2* | 34 | NA | 430 | 460 | 470 | 410 | NA |
| *3* | 12 | 550 | 690 | 630 | 530 | 440 | NA |
| *4* | 76 | 1610 | 1230 | 1180 | 1200 | 870 | 1460 |
| *5* | 93 | 830 | 620 | 850 | 500 | 650 | 510 |
| *6* | 68 | 490 | 550 | 480 | 600 | 740 | 500 |
| *7* | 35 | 590 | 570 | 660 | 470 | 360 | NA |
| *8* | 44 | 770 | 390 | NA | 660 | 320 | NA |

## S7: Effect of sub-sampling on BCR statistics

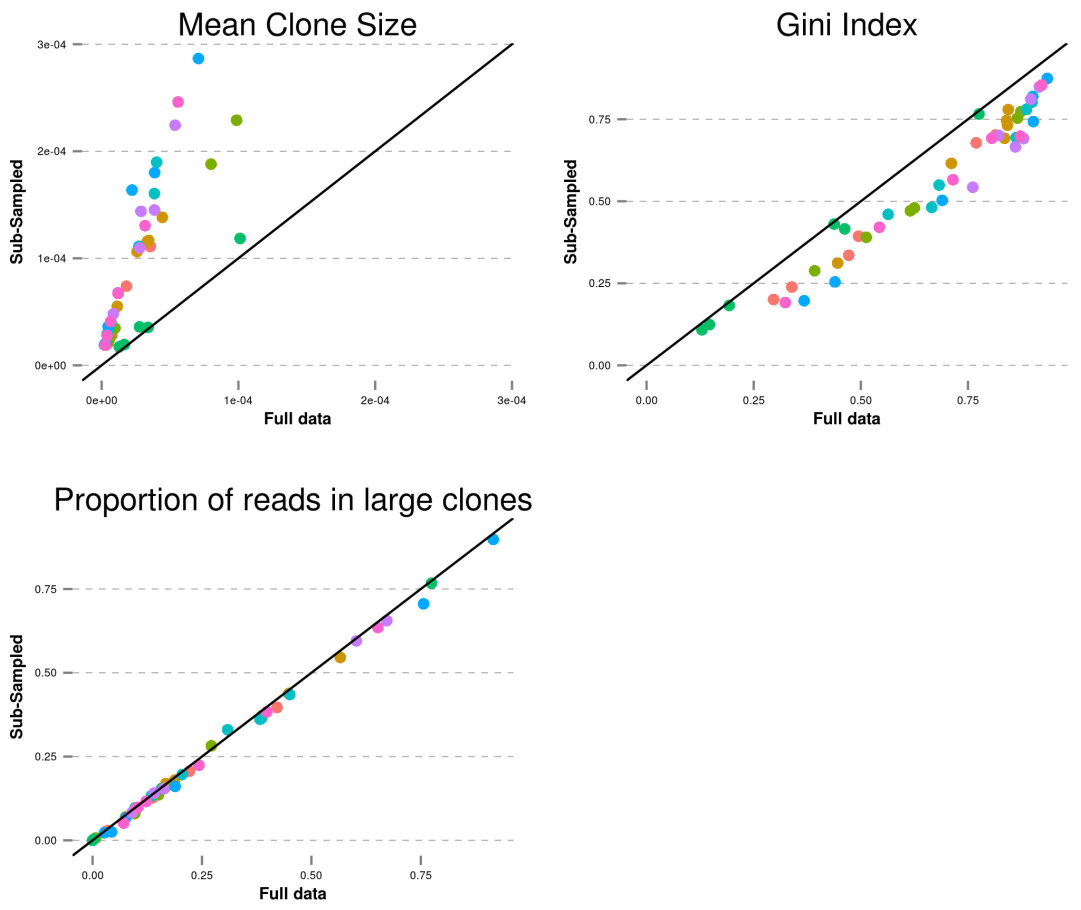


*S7*: BCR statistics of HIV+ patients calculated from full data versus data sub-sampled data. Read depth of full data had a median of 567,936 and a range of 74,861 to 1,040,936 reads per patient per time point. Sub-sampled data were randomly sub-sampled to a depth of 70,000 reads per patient per time point. Dots are coloured by patient.
